# Generation of multicolor banding probes for chromosomes of different species

**DOI:** 10.1186/1755-8166-6-6

**Published:** 2013-02-04

**Authors:** Nadezda Kosyakova, Ahmed Basheer Hamid, Arunrat Chaveerach, Krit Pinthong, Pornnarong Siripiyasing, Weerayuth Supiwong, Svetlana Romanenko, Vladimir Trifonov, Xiaobo Fan

**Affiliations:** 1 Jena University Hospital, Friedrich Schiller University, Institute of Human Genetics, Kollegiengasse 10, Jena, D-07743, Germany; 2 Department of Biology Faculty of Science, Khon Kaen University, 123 Moo 16 Mittapap Rd., Muang District, Khon Kaen, 40002, Thailand; 3 Faculty of Science and Technology, Surindra Rajabhat University, 186 Moo 1, Maung District, Surin, 32000, Thailand; 4 Faculty of Science and Technology, Rajabhat Maha sarakham University, 80 Nakonsawan Rd., Talad, Maung District, Maha sarakham, 44000, Thailand; 5 Institute of Molecular and Cellular Biology, Lavrentev Str. 8/2, Novosibirsk, 630090, Russia

**Keywords:** Chromosome painting, FISH-banding, Multicolor banding, Chromosome microdissection

## Abstract

**Background:**

The multicolor banding (MCB/mBAND) technique provides a unique opportunity to characterize intrachromosomal rearrangements and to determine chromosomal breakpoints. Until recently, MCB probes have only been available for human and some murine chromosomes. Generation of MCB probes for chromosomes of other species, useful and required in many cytogenetics research fields, was limited by technical difficulties. MCB probes are established by chromosome microdissection followed by whole genomic DNA amplification. However, unambiguous identification of the target chromosome is required for MCB-probe establishment. Previously proposed protocols suggested G-banding staining or preliminary FISH with whole chromosome paints (WCP) as methods to identify the chromosome of interest.

**Results:**

Here we present a complete workflow for MCB probe generation for those cases and species where chromosome morphology is too challenging to recognize target chromosomes by conventional methods and where WCP probes are not available. The workflow was successfully applied for murine chromosomes that are difficult to identify unambiguously. Additionally, we showed that glass-needle based microdissection enables establishment of a whole set of WCP paints by microdissection of individual chromosomes of a single metaphase

**Conclusions:**

The present method can be applied for generation of whole or region-specific DNA probes for species, where karyotyping of G-banded chromosomes is challenging due to similar chromosome morphology and/or chromosome banding patterns.

## Background

The identification of chromosomes and chromosomal subregions can be a challenging task. While in clinical cytogenetics and in many mammalian species the available staining methods provide informative banding patterns along the chromosomes, there are many more species in which no such chromatin-related patterns of alternated light and dark bands can be induced; for example, G-banding is only reliable in higher vertebrates [1]. Introduction of FISH (fluorescence *in situ* hybridization) approaches was quite helpful for further progress; however, for many species there is a lack of available DNA probes. Glass needle-based microdissection of chromosomes can be applied to establish chromosome specific probes of different species [2,3]. Besides whole chromosome painting probes (WCP), partial chromosome painting (PCP) probes can also be extremely helpful for the characterization of chromosomes of closely related species and their evolutionary relations to each other. Furthermore, a set of PCPs can serve as а base for the so-called multicolor banding (MCB/mBAND) technique, originally proposed in 1999 [4] for human chromosomes. As MCB probes can unambiguously determine pericentric and paracentric inversions and map the breakpoints, they were immediately recognized to be a useful tool for studying chromosomal evolution. Human MCB probes have been successfully applied to characterize in detail karyotypes of *Gorilla gorilla*[5], *Hylobates lar*[6] and *Pan paniscus*[7]. However, application of human MCB probes on chromosomes of evolutionarily distant species is challenging, and often not feasible. This created the necessity of generating MCB probes for the chromosomes of other species, potentially interesting from the cytogenetic point of view. The first attempt to generate FISH-banding probes for non-human chromosomes was made by our group in 2002, when multicolor banding probes for mouse chromosomes 3, 6, 18, 19 and X were established; published in 2006 [8]. To distinguish murine/non human multicolor banding probes from the previously established MCB probes for human chromosomes, it was suggested to use lowercase letters, as opposed to capital letters, in the abbreviation – “mcb” (the same was suggested for non-human WCP probes – “wcp”). In 2003 another group also established a similar probe set for mouse chromosome 11 [9]. However, no further mouse mcb generation experiments followed. This was partly due to technical difficulties in generating region specific DNA libraries/probes to be incorporated into mcb probe mixes.

PCP probes for MCB probe mixes are generated conventionally by glass needle microdissection [4,10,11], which allows precise isolation of the target chromosome region that can be as small as a single G-band. The absolutely necessary condition for chromosome microdissection is the possibility to identify the target chromosome unambiguously. The conventional microdissection protocol applies trypsin-Giemsa staining to distinguish chromosomes and visualize chromosome bands. Therefore, the quality of suspension is a crucial factor: chromosomes must be of reasonable length and metaphases should be well spread. In many cases, for example in tumor samples, karyotyping and identification of a chromosome/chromosome region to be microdissected can be quite challenging. The same is also true for the specimens from other species with chromosomes of similar morphology and banding patterns. About a decade ago, a so called FISH-microdissection (FISH-MD) technique was proposed [12]. It enabled identification of a target chromosome by FISH with whole chromosome paint (WCP) probes and its immediate dissection from the same metaphase. This, however, requires the availability of WCP probes for the studied specimen. Although WCP probes are commercially available for human, mouse and rat chromosomes, and WCP libraries were established for many other species using flow-sorting followed by whole genome amplification, these WCP probes are not available for all potentially interesting species/chromosomes. Here we propose a complete workflow to generate wcp and pcp probes, as well as mcb probe sets when chromosome morphology is too challenging to recognize target chromosomes by conventional methods and when other probes are not accessible.

## Results and discussion

**Figure 1:**
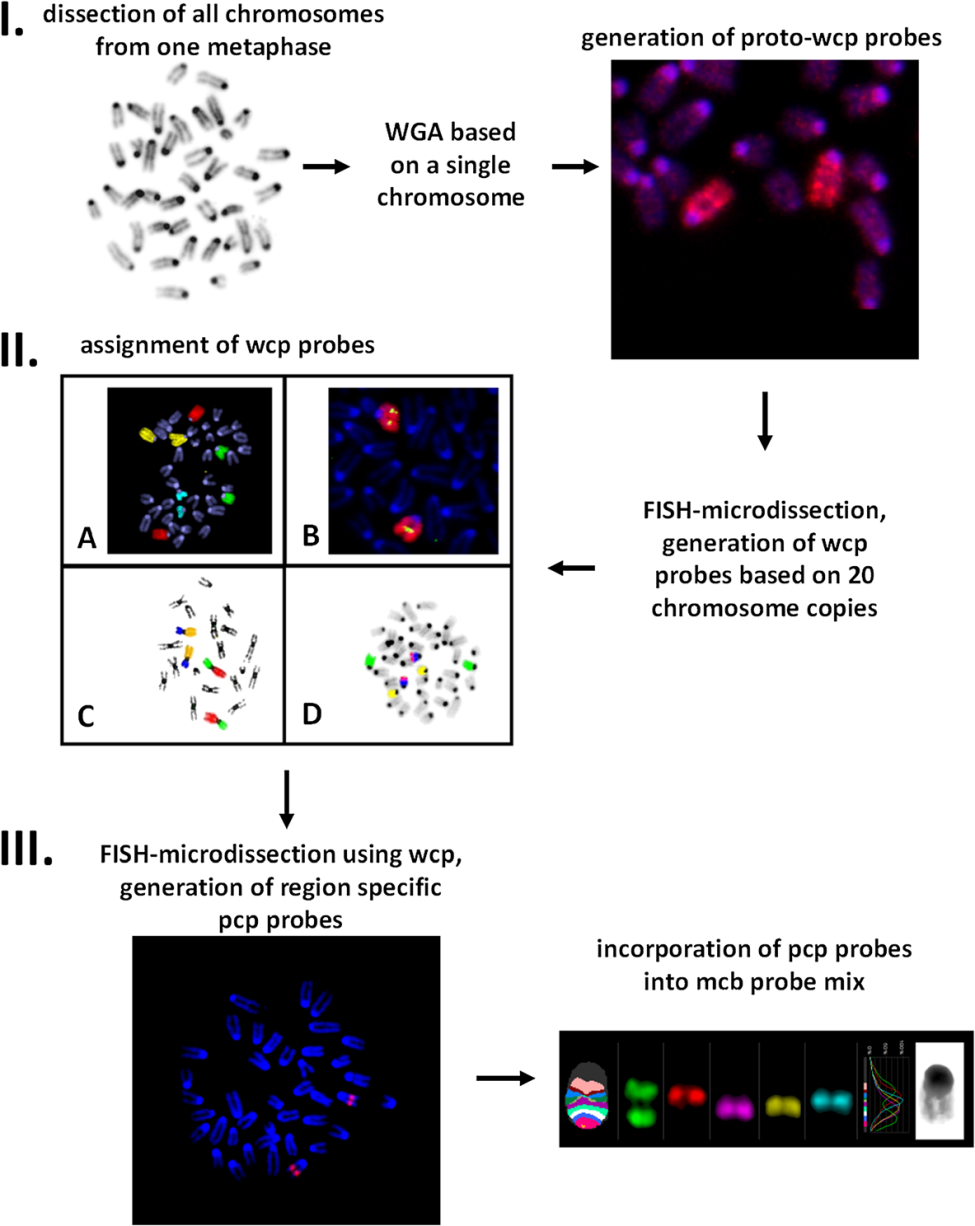
**mcb probe generation workflow, applicable to other species as well. I** Murine metaphase stained with Giemsa. All chromosomes from this metaphase were dissected one by one and collected into separate micropipettes. Original amplification based on a single chromosome template resulted in proto-wcp probes. **II** Generation of murine wcp probes and their attribution. “Doubles” corresponding to the same pair of homologue chromosomes were excluded by several rounds of co-hybridization (**A**). Chromosome-specific libraries were assigned by co-hybridization with available BAC probes (**B**), available murine pcp probes (**D**), or by applying them onto metaphase spreads of WMP2 cell line (**C**). **III** Generation of murine pcp paints and their incorporation into mcb probe mixes. Here, reverse-FISH of the pcp from murine chromosome 9 corresponds to FISH-MD experiment in Additional file 1: Video 1. Fluorochrome profile and pseudocolor banding pattern resulting after application of murine mcb 9.

The whole workflow for generating mcb probes for those cases, where chromosome morphology makes chromosome identification complicated, is presented in Figure 1. Mouse model was chosen for several reasons. Mouse chromosomes are all telocentric; this fact and difficulties in G- and R-banding complicate karyotyping of mouse strains and evaluation of stability of murine cell lines. Besides, the challenges in murine chromosome banding pattern recognition along with high rates of karyotype rearrangements complicate the cytogenetic evolutionary studies among rodents, which are regarded to be very good models for investigating karyotype evolution in vertebrates. Therefore there was an urgent necessity for murine mcb paints.

**Figure 2:**
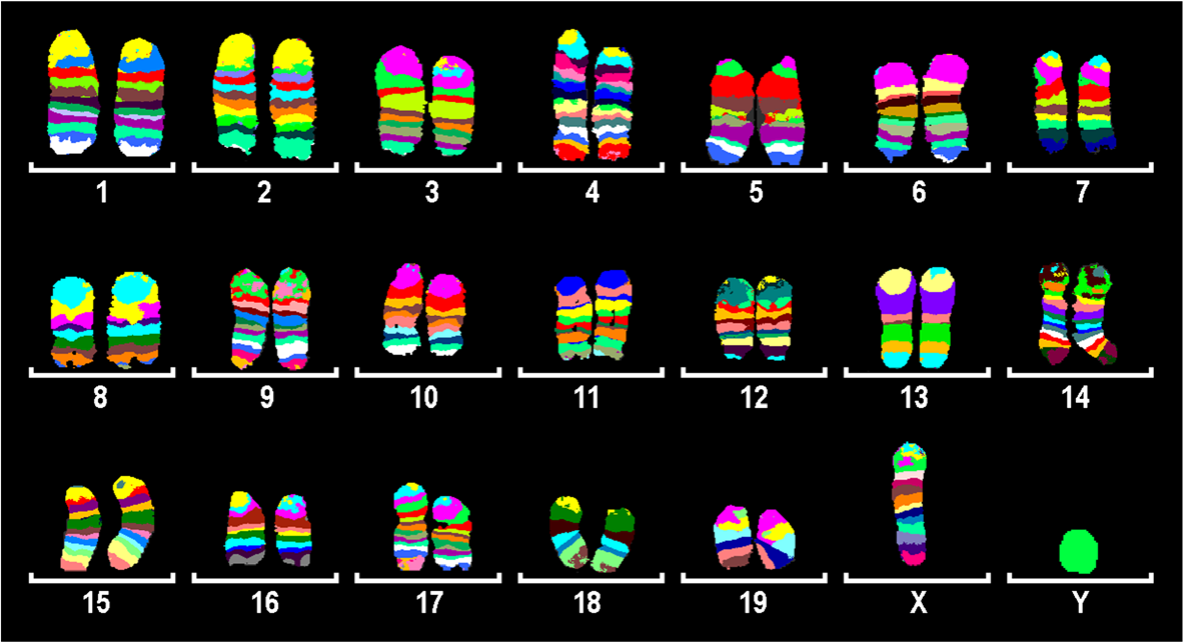
**Combined image of pseudocolor pattern for all 19 mouse autosomes and for the X chromosome.** Two homologue chromosomes are presented each, apart from gonosomes.

At first we aimed to establish wcp probes for all mouse chromosomes to be later used in FISH-MD experiments. The strategy we applied was to dissect all chromosomes of the same metaphase one by one and to collect them into separate micropipettes, containing chromosome collection buffer (30% glycerol, 10 mM Tris/HCl, pH 7.5, 10 mM NaCl, 0.1% SDS, 1 mM EDTA, 0.1% Triton X-100, 1.44 mg/ml proteinase K). The collection buffer with a single dissected chromosome was then transferred into a tube with 5 μl of PCR-grade water. Further amplification was done using GenomePlex® Single Cell Whole Genome Amplification Kit (Sigma-Aldrich). The resulting 40 proto-wcp probes corresponding to 40 murine individual chromosomes were based on the amplification of DNA from a single dissected chromosome. These proto-wcp paints produced weak, but identifiable signals on one pair of chromosomes upon reverse-FISH back onto mouse metaphases (apart from two probes which obviously corresponded to X and Y chromosomes and resulted in staining only a single chromosome in a male murine cell, each). These proto-wcp probes were further used in FISH-MD experiments to generate wcp probes based on 20 dissected chromosomes per probe. Collection of multiple chromosomes aimed to improve the representation of all chromosomal regions in the generated library and to exclude the possibility of loss of any of the fragments while dissecting or collecting them into micropipettes. After this second round of microdissection 40 murine wcp paints were obtained. But as they were generated “blindly” it was not known which wcp corresponded to which chromosome. To assign these wcp probes, we performed several rounds of co-hybridization. Initially, we co-hybridized different wcp probes labeled with different fluorochromes onto control mouse metaphases to exclude the identical probes corresponding to the same pair of homologous chromosomes. When the doubles were excluded we got 19 unique wcp probes for autosomes and wcp probes for the X and Y chromosomes (which were identified earlier). To detect which wcp corresponded to which chromosome we have used several different approaches: 1) wcp probes were applied onto metaphases from WMP2 cell line known to contain easily identifiable metacentric fusion chromosomes characterized before by G-banding and FISH [8,13]; 2) wcp probes were co-hybridized with murine BAC probes available in our laboratory 3) wcp probes were co-hybridized with previously obtained murine region specific probes [8]. After all wcp probes were successfully attributed, they were further used in FISH-MD experiments to generate region-specific pcp paints required for mcb probe sets. An example of such FISH-MD with murine wcp probe for chromosome 9 can be seen in Additional file 1: Video 1. As chromosomes were not counterstained and visualized by phase contrast, chromosome regions were dissected based only on chromosomal size/proportions (for example, the distal or the proximal one third/fourth part of the chromosome was dissected). For every pcp library 15–20 copies of chromosome fragments were used as a template. Overall, 115 pcp libraries covering all 19 murine autosomes and the X chromosome were established. Each pcp probe was tested by reverse-FISH on control mouse metaphases, and assigned cytogenetically based on inverted DAPI bands. Three to eight (depending on chromosome’s size) overlapping pcps correspond to each chromosome. The changing fluorescence intensity ratios along the chromosomes were used by isis mBAND software (MetaSystems Hard & Software GmbH, Altlussheim, Germany) to assign different pseudocolors to specific chromosomal regions (Figure 2). The obtained mcb paints for all murine chromosomes were already successfully applied to characterize the widely used NIH3T3 cell line [14].

Hence, in spite of the fact that it was not possible to reliably identify chromosomes in mouse metaphase spreads based on GTG-banding, we have successfully generated mcb probe sets for all 19 murine autosomes and the X chromosome. The only chromosome, for which we have failed to establish an mcb mix, was the Y-chromosome – all pcp probes for the Y-chromosome resulted in staining of the entire chromosome. This fact might be due to the enormously expended ampliconic portion of the mouse Y chromosome, taking up to 95% of the chromosome [15], so all of the dissected DNA libraries contained repeat units which might have been preferentially amplified during the original amplification step.

We believe that the proposed workflow can be applied for chromosomes of other species as well in order to generate wcp, pcp and mcb probe sets. The approach of “blind” microdissection of all chromosomes of one single metaphase is especially helpful for obtaining chromosome-specific DNA libraries for the cases when chromosome isolation by flow sorting is hampered by similar size of chromosomes.

One of the problems one may encounter while establishing wcp or pcp paints can be the selection of suitable DNA amplification method. For many years DOP-PCR with 6MW primer was a method of choice for amplifying microdissected material, and it is still conventionally used for amplification of human microdissected chromosomes. The anti-6MW DOP-PCR primer was proposed later [16] to help to select against mouse satellite DNA and to facilitate successful amplification of murine chromosomes. Lately several new commercially available whole genome amplification methods were tested for amplifying microdissected chromosomes, and GenomePlex® Single Cell Whole Genome Amplification Kit (Sigma-Aldrich) linker adapter PCR approach was declared to have higher rate of successful amplification [17]. We applied GenomePlex® Single Cell Whole Genome Amplification Kit for amplification of single chromosomes and subsequent generation of wcp probes to avoid the preferential amplification of satellite DNA. Though the method worked well in general, we experienced a significant reduction in the kit efficiency over time in spite of proper storing conditions. It should be also kept in mind that the amount of DNA used as a template after microdissection is dramatically lower than the one required by most of the commercially available kits. For the pcp libraries generation we have tried DOP-PCR with both 6MW and anti-6MW primers as well as GenomePlex® Single Cell Whole Genome Amplification Kit. All approaches resulted in a reasonable quality of obtained probes as tested by reverse-FISH. However, the DOP-PCR seemed to be a more robust and reliable option, which also enabled further re-amplification and PCR labeling of generated probes by the same primer. To re-amplify and to label the probes derived by GenomePlex® Single Cell Whole Genome Amplification Kit one may require additional amounts of the Amplification Master Mix component of the kit.

Application of mcb probes requires prehybridization with Cot1 DNA for the suppression of repetitive elements to block non-specific hybridization. The source of Cot1 DNA for the species of interest should be available, as Cot1 DNA is essential for the FISH-MD experiments with wcp paints, as well as for reverse-FISH tests and mcb probe applications. Due to the fact that all DNA which is dissected and collected into a micropipette is subjected to amplification, we believe it is also important to reduce the amount of Cot1 DNA used in FISH-MD experiments to avoid the excessive amplification of repetitive elements in the generated DNA library (the dissected fragments contain DNA from the hybridized wcp probe). The exact amount of Cot1 DNA used in FISH-MD and in reverse-FISH should be determined experimentally.

Though glass needle based microdissection requires experience and is time consuming, it is still a method of choice for generating pcp paints as it works finer than other techniques and enables precise dissection and collection of target chromosome regions. It is even possible to dissect several different pcp libraries corresponding to different regions from the same chromosome, thus saving chromosome material. We were able to attribute “blindly” generated murine wcp paints by co-hybridizing them with other region specific and BAC probes available in our laboratory. For the chromosomes of other species these kinds of probes can be simply not available; we suggest that in such cases wcps can be identified by painting the previously G-banded chromosomes.

## Conclusions

We have demonstrated that generation of mcb probes is possible even in cases where chromosome morphology complicates chromosome recognition. The proposed approach of “blind” microdissection of all chromosomes of the same metaphase can be also helpful for establishing chromosome-specific DNA libraries for the species which chromosomes cannot be flow sorted due to their similar size.

FISH-banding methods and mcb technique especially, open new opportunities for evolutionary cytogenetics studies. Recent application of some murine mcb paints in nine rodent species [18] has proved to be highly efficient in detecting cryptic aberrations and intrachromosomal rearrangements, pointed the evolutionary conserved breakpoints and helped to reveal previously unrecognized segments of homology, which were not identified by G-banding, neither detected by previous experiments with murine wcp probes. However, hybridization efficiency drops down as phylogenetic distance increases, affecting signal intensities and stability of pseudocolor bands. Therefore the elaboration of mcb probes for chromosomes of other species is considered to be necessary. The proposed workflow of generation of mcb probes can be applied to chromosomes of any species which metaphase spread preparations are available; thus enabling molecular cytogenetic characterization and comparison of chromosomes of different species, revealing their phylogenomic relationships.

## Methods

### Chromosome suspensions and microdissection

Murine chromosome spreads of different origin were used for microdissection and FISH: mouse embryonic and adult fibroblasts cultures, short-term cultivation of murine spleen tissue and WMP2 cell line [13]. WMP2 cell line was kindly provided by Dr. Hameister (Ulm, Germany). Cell cultivation and fixation followed the standard conditions. In brief, fibroblast cultures were cultivated in DMEM-, and murine spleen tissue and WMP2 cell line in RPMI-1640-medium with 10% fetal calf serum. Prior to harvesting cells were incubated with 0.12 μg/ml colcemid solution, and then harvested using hypotonic treatment and fixation in 3:1 methanol/glacial acetic acid. All obtained preparations were checked for the number and morphology of chromosomes prior to use. Chromosome microdissection and collection of dissected fragments followed the previously published protocol [11]. FISH-MD experiments were done according to [12].

### Amplification of microdissected material and probe preparation

Amplification of microdissected DNA was carried out either with DOP-PCR [19], or with GenomePlex® Single Cell Whole Genome Amplification Kit (Sigma-Aldrich). Two different types of primers were used for conducting DOP-PCR reaction: the 6-MW (5^′^-CCG ACT CGA GNN NNN NAT GTG G-3^′^) and the anti-6MW (5^′^-CCG TGA GCT CNN NNN NTA CAC C-3^′^) primer [16]. 3 μl of amplification product were run on a 2% agarose gel to check for the presence a clearly visible smear with an average size of 0.2-1.0 Kb (evidence of successful amplification). After original amplification all generated DNA libraries were subjected to one or two rounds or re-amplification, and then labeling – either by PCR with 6MW/anti-6MW primer [11], or by PCR based on the components of the Single Cell Whole Genome Amplification Kit (depending on the original choice of amplification). Probe preparation followed standard protocols [20]. Before hybridization all probes were mixed with the appropriate amount of mouse Cot-1 DNA® (Invitrogen).

Mouse BAC probes were purchased from the Children’s Hospital Oakland Research Institute (CHORI), Oakland, USA. All BAC clone DNA was isolated, PCR-amplified and labeled as described [21].

### FISH and image analysis

Hybridization, post-hybridization washes and detection steps were done as described previously [20]. Image acquisition was performed using an Axioplan II microscope (Carl Zeiss Jena GmbH) equipped with filter sets for DAPI, FITC, TR, Cy3 and Cy5. Image analysis was done with the help of isis mBAND software (MetaSystems Hard & Software GmbH, Altlussheim, Germany), which is essential for MCB evaluation as no other image analyzing system provides the possibility to evaluate fluorochrome profiles and to create pseudocolor banding along the chromosomes. All generated probes were first tested on murine metaphase spreads prior to use/incorporation into probe mixes to evaluate the quality and to exclude contamination. Region specific probes were mapped cytogenetically based on the inverted DAPI banding pattern of chromosomes.

## Abbreviations

DNA: Deoxyribonucleic acid; PCR: Polymerase chain reaction; DOP-PCR: Degenerate oligonucleotide-primed PCR.

## Competing interests

The authors declare that they have no competing interests.

## Authors’ contributions

NK conceived the study, performed microdissection experiments and drafted the manuscript. ABH, KP, PS, WS and SR performed microdissection and FISH experiments. AC, VT and XF participated in the study design and protocol elaboration, and helped to draft the manuscript. All authors read and approved the final manuscript.

## Supplementary Material

Additional file 1 **Video 1.** FISH-MD experiment. Murine chromosome 9 is identified by FISH with wcp 9 probe labeled with Spectrum Orange-dUTP (pointed with a white arrow). Microdissection is done using phase-contrast imaging as chromosomes are not counterstained. After chromosome region is dissected, it is transferred into a micropipette containing collection buffer. (AVI 16311 kb) Click here for file
